# Interactions between Rice Resistance to Planthoppers and Honeydew-Related Egg Parasitism under Varying Levels of Nitrogenous Fertilizer

**DOI:** 10.3390/insects13030251

**Published:** 2022-03-01

**Authors:** Ainara Peñalver-Cruz, Finbarr G. Horgan

**Affiliations:** 1 Institut de Génétique, Environnement et Protection des Plantes (IGEPP), Institut National de Recherche pour l’Agriculture, l’Alimentation et l’Environnement (INRAE), Institut Agro, Université de Rennes, CEDEX, 49045 Angers, France; ainara.penalver@agrocampus-ouest.fr; 2 International Rice Research Institute, Makati 1226, Metro Manila, Philippines; 3 EcoLaVerna Integral Restoration Ecology, Bridestown, Kildinan, T56 P499 County Cork, Ireland; 4 Escuela de Agronomía, Facultad de Ciencias Agrarias y Forestales, Universidad Católica del Maule, Casilla 7-D, Curicó 3349001, Chile; 5 Centre for Pesticide Suicide Prevention, University/BHF Centre for Cardiovascular Science, University of Edinburgh, Edinburgh EH16 4TJ, UK

**Keywords:** *Anagrus* spp., *BPH32* gene, conservation biological control, honeydew, host plant resistance, integrated pest management, nectar, *Nephotettix* spp., *Nilaparvata lugens*, *Sogatella furcifera*

## Abstract

**Simple Summary:**

Planthopper outbreaks in rice are associated with excessive fertilizer applications. Public research has focused on developing resistant rice to combat these outbreaks. However, to preserve ecosystem resilience, natural enemy efficacy should be maintained on resistant and susceptible rice. We examined the impact of egg parasitoids on planthoppers (*Nilaparvata lugens* (Stål) [BPH] and *Sogatella furcifera* (Horváth) [WBPH]) and a leafhopper (*Nephotettix virescens* (Distant) [GLH]) in field plots of resistant (IR62) and susceptible (IR64) rice under low and high nitrogen. GLH and WBPH were more abundant in low-nitrogen plots during dry (GLH) and wet (GLH, WBPH) season sampling at an early crop stage. GLH were also more abundant on IR64. Parasitoids killed between 24 and 52% of planthopper eggs during exposures in trap plants. Parasitism by *Oligosita* and *Anagrus* wasps was higher on IR64 (BPH eggs) and in high-nitrogen plots (*Oligosita* spp. on BPH and WBPH eggs; *Anagrus* spp. on BPH eggs). Parasitism by *Anagrus* spp. was associated with the presence of honeydew and was highest where honeydew was derived from BPH feeding on IR62; these effects were only observed under high nitrogen. Results suggest that honeydew from IR62 favors parasitoids when plants are most vulnerable (i.e., under high nitrogen), thereby countering nitrogen-induced declines in host resistance.

**Abstract:**

Host plant resistance is the most researched method for the management of planthoppers and leafhoppers in tropical rice. For optimal effects, resistance should be resilient to fertilizer inputs and work in synergy with natural enemies. In field plot experiments, we examined how rice resistance and fertilizer inputs affect mortality of planthopper and leafhopper eggs by hymenopteran parasitoids. We used IR62 as a variety with resistance to *Nilaparvata lugens* (Stål) [BPH], *Sogatella furcifera* (Horváth) [WBPH] and *Nephotettix virescens* (Distant) [GLH], and IR64 as a susceptible control. The herbivores were more abundant during wet season sampling in low-nitrogen plots. During this study, parasitoids killed between 31 and 38% of BPH eggs and 24 and 52% of WBPH eggs during four days of field exposure. Parasitism, mainly due to *Oligosita* spp., was generally higher in high-nitrogen and IR64 plots. Similar densities of eggs in exposed plants suggest that these trends were mediated by semiochemicals and therefore support the Optimal Defense Hypothesis. Honeydew from BPH on IR62 had more xylem-derived wastes than honeydew on IR64. We applied honeydew from both varieties to sentinel plants. Parasitism by *Anagrus* spp. was higher on plants of either variety treated with honeydew derived from IR62; however, the effect was only apparent in high-nitrogen plots. Results suggest that *Anagrus* spp., by responding to honeydew, will counter the nitrogen-induced enhancement of planthopper fitness on resistant rice.

## 1. Introduction

Rice is the main staple food for over 50% of the world’s population and is mainly produced in Asia [1]. Much of that production occurs in intensified lowland agroecosystems [1,2]. Pressures to increase rice production to feed Asia’s growing population have led to further intensification through the expansion of rice production into previously natural areas, the adoption of high-yielding rice varieties, a high use of chemical fertilizers, and an increase in the frequency of pesticide applications [3]. However, high fertilizer inputs increase the susceptibility of rice to several insect herbivores, and particularly to phloem-feeding herbivores [4,5,6]. Furthermore, several studies have associated insecticides with increases in the frequency of planthopper and leafhopper outbreaks across Asia [7,8,9,10,11,12], with insecticide-related mortality of natural enemies representing a major mechanism underlying these outbreaks [9,13,14,15,16]. Sustainable pest management strategies that maintain or enhance natural enemy abundance and activities, particularly under a high use of inorganic fertilizers, are therefore required for intensified rice production systems.

Recent public research has focused on two main strategies for planthopper and leafhopper management. Firstly, research into the development of planthopper- and leafhopper-resistant rice varieties has begun to use novel molecular tools to search for and incorporate resistance genes from traditional varieties and wild rice species into modern, high-yielding rice varieties [17]. To date, over 60 genes for resistance to rice planthoppers and over 20 genes for resistance to leafhoppers have been identified. A large number of rice varieties with resistance have been tested, or released to farmers [18,19]. Secondly, because insecticides were associated with a decline in regulation services provided by the natural enemies of rice herbivores, research into conservation biological control has gained traction in several Asian countries [20,21,22,23,24]. In particular, researchers have investigated possibilities to incorporate functional plants into rice ecosystems to augment natural enemy abundance and diversity and to improve natural enemy efficacy in regulating key rice herbivores. Research from China, Vietnam, Thailand, the Philippines, Bangladesh and Cambodia has indicated that, by planting flowering crops on rice levees (also known as bunds), the abundance and diversity of natural enemies can be increased [20,21,22,23,24,25]. For example, nectar produced by bund flowers is thought to sustain the free-living stages of egg parasitoids [26]. 

Despite recognizing the importance of supplementary foods for natural enemies, little is known about the effects of honeydew on parasitism in rice ecosystems. Planthoppers and leafhoppers produce honeydew as a waste during feeding. Honeydew is excreted through the anus and often coats the host plant surface close to feeding locations (i.e., mainly around the stem base when rice is attacked by planthoppers) [27]. The composition of honeydew is strongly influenced by the rice variety on which planthoppers or leafhoppers feed, and particularly on whether the variety is resistant or susceptible to planthoppers [28,29,30]. For example, honeydew produced from planthoppers feeding on susceptible rice is mainly derived from phloem feeding, whereas planthoppers on resistant rice often produce honeydew that is predominantly derived from xylem feeding and is, therefore, likely to contain fewer nutrients [30]. Furthermore, honeydew composition is affected by crop fertilizer levels; for example, planthoppers feeding on rice grown in high-nutrient soils increase their phloem-based feeding (compared to xylem-based) and produce larger quantities of honeydew [31]. Honeydew represents a significant nutrient source for the parasitoids of phloem-feeding herbivores, such as aphids, in other cereal systems [32,33,34,35]; however, to our knowledge, the role of honeydew as a food for the parasitoids of rice planthoppers or leafhoppers has not yet been studied. 

A small number of studies have quantified planthopper egg mortality due to parasitoids in fields with different rice varieties or fertilizer rates [5,22,36,37,38]. These different field experiments have produced inconsistent results [22,37,38] that, to some extent, may be due to the many varieties used in the studies. In particular, the semiochemicals derived from different varieties of rice during herbivore attacks likely vary in their effectiveness as synomones that ‘call in’ defending parasitoids [37]. However, semiochemical profiles might also change in response to resource availability (i.e., nitrogen, light, and water), often with plants increasing their defenses where conditions are most favorable for growth (i.e., Optimal Defense Hypothesis [39]). These ideas suggests that semiochemicals could be optimally matched with biocontrol agents through plant breeding or nutrient management to increase parasitoid-mediated mortality of insect pests in intensified field crops [40,41,42,43]. Where parasitoids also gain a sugar source by feeding on honeydew, the correct blend of semiochemicals may interact with honeydew quality (i.e., available nutrients, consistency, or related kairomones) to further increase parasitism rates. 

In the present study, we examine interactions between rice resistance to planthoppers (*Nilaparvata lugens* (Stål) (BPH) and *Sogatella furcifera* (Horváth) (WBPH)) and a leafhopper (*Nephotettix virescens* Distant (GLH)) and mortality due to egg parasitoids under varying soil nitrogen regimes. We examined whether parasitism of planthopper eggs was greater in fields with high-nitrogen applications as predicted by the Optimal Defense Hypothesis and suggested by evidence of semiochemical lures for egg parasitoids. We also hypothesized that parasitoids would respond to differences in the composition of planthopper honeydew by parasitizing a greater number of eggs in fields of susceptible rice and under higher levels of soil nitrogen (i.e., where honeydew has a higher nutrient content and is less watery). Previous studies have indicated that egg parasitoids have increased fitness (e.g., female longevity [26]) after feeding on high-nutrient solutions, such as nectar and honeydew (compared to water), ultimately increasing parasitoid effectiveness as biological control agents [44,45,46]. We therefore predicted that higher proportions of planthopper eggs would be parasitized in fields of susceptible rice and under relatively high-nitrogen fertilizer inputs, at least partially in response to honeydew on the plant surface. We tested these predictions by exposing sentinel plants (susceptible and resistant to planthoppers) with planthopper eggs in replicated field plots planted with a highly resistant (IR62) and a susceptible (IR64) rice variety under two levels of nitrogenous fertilizer. We also manipulated honeydew coatings on sentinel plants by adding or removing honeydew, or by transferring honeydew between resistant and susceptible plants. We discuss our results in terms of better integrating host plant resistance into conservation biological control for rice planthoppers in tropical Asia. 

## 2. Materials and Methods

### 2.1. Herbivore Species

We conducted experiments with the brown planthopper (BPH), the whitebacked planthopper (WBPH), and the green leafhopper (GLH). To prepare sentinel plants, we used gravid females of each species taken from colonies maintained at the International Rice Research Institute (IRRI) in the Philippines. The GLH colony was initiated in 2008 with approximately 500 individuals collected from rice fields in Laguna Province, Luzon. The BPH and WBPH colonies were initiated in 2009, each with 500 individuals collected from the same fields in Laguna Province. Colony initiation and maintenance were the same for each colony. The founder populations were placed in separate wire mesh cages of 120 × 60 × 60 cm (H × W × L) under screenhouse conditions (temperatures = 25–37 °C; relative humidity = 70–90%; 12 h:12 h, D:N). The planthoppers and leafhoppers were continuously reared on a susceptible rice variety (variety = Taichung Native 1 [TN1]) at ≥30 days after sowing (DAS). Feeding plants were changed every one or two weeks. BPH from Laguna are virulent against rice with resistance derived from the *Bph1*, *bph2*, *bph5*, *bph7*, *Bph18*, *BPH25* and *BPH26* genes; WBPH are virulent against *WbphM1* and *WbphM2* [47]. Colonies had been maintained for between 35 and 45 generations before use in the experiments.

### 2.2. Plant Materials

We used two rice varieties in our experiments. IR62 is a highly resistant, ‘modern’ variety that was released by IRRI in 1984. The variety has high resistance to BPH populations from South and Southeast Asia [48]. IR62 is also resistant to WBPH and GLH in the Philippines [47]. However, resistance against WBPH is predominantly expressed after maximum tillering [31]. Resistance in the variety was derived from the Indian landrace PTB33 and is likely associated with the *Bph3* locus, which contains the *Bph3* and/or *Bph32* resistance genes [49,50]. Although several varieties with PTB33 as a resistance donor were released by IRRI, IR62 appears to have particularly strong resistance to planthoppers and has maintained its resistance for over 30 years [49]. IR64 was used as a susceptible control variety in our experiments. IR64 was released by IRRI in 1985. Since then, the variety has become a mega-variety in South and Southeast Asia, largely due to its high-quality rice grain [51]. Because of its popularity, several beneficial traits, including flood tolerance, have been incorporated into IR64 using marker-assisted selection [51]. IR64 contains the *Bph1* gene for resistance, as well as quantitative resistance and tolerance [52]. However, widespread exposure of the *Bph1* gene over three decades throughout Asia has resulted in a high proportion of planthopper populations with virulence against the gene, such that the variety is now susceptible to BPH [48]. Seed of both varieties was acquired through the germplasm bank at IRRI. Recent greenhouse studies have verified that, compared to IR64, the resistant variety IR62 reduces planthopper population or biomass build-up over successive generations after initial infestation [53]. Furthermore, research using field cages indicates that IR62 prevents ‘hopperburn’ (i.e., planthopper-induced death of rice plants) [54].

### 2.3. Field Plot Design

Field experiments were conducted during the 2013 dry (DS) and wet (WS) seasons at the Ecological Function Experimental Platform (EcoFun) of the IRRI Experimental Field Station in Los Baños, Philippines. The EcoFun plots have been described in detail by Horgan et al. (2019) [22]. At the time of the experiments, the platform consisted of six rice fields divided into 18 plots of 33 × 12.5 m (L × W). Separate subirrigation channels were installed around each plot. These connected to the main field canals for irrigation and drainage, but prevented leakage of nutrients between adjacent fields or between plots within each field. The field plots were treated with one of three nitrogen levels each season. These were: zero added nitrogen (0 kg N ha^−1^), 60 kg N added ha^−1^ and 150 kg N added ha^−1^. Nitrogen (ammonium sulfate) was applied as four top dressings (basal, mid-tillering, panicle initiation and at one week before flowering). Solophos, muriate of potash and zinc were applied basally with the ammonium sulfate. The present study was conducted only using the 0 kg and 150 kg N ha^−1^ plots (i.e., 12 plots). During 2013, these 12 plots were each divided into four subplots of 8.25 × 11 m, with two subplots each planted with either IR62 or IR64 (varieties were assigned randomly to subplots). Seed was initially sown to low-density, dry seedbeds in a screenhouse, and the ‘seedlings’ (early-tillering stage) were transplanted, 28 days later, as one plant per hill to the puddled field plots. Hills were spaced at a distance of 10 cm in half of the subplots and 20 cm in the other half (i.e., IR62 and IR64 plots were each planted at high and low density). For the purposes of this research, experiments were conducted only in the low-density subplots. Furthermore, sampling was conducted at the maximum possible distance from bund vegetation. No pesticides were used in the fields at any time during the experiments. Light weeding was conducted by hand at the beginning of each cropping season. All experiments described in this study were conducted during the rice tillering stage of each crop (i.e., <45 days after transplanting [DAT]; late February in the DS, early August in the WS), which corresponded with the time of maximum parasitoid activity in the fields (Horgan, unpublished data).

### 2.4. Sampling of Free-Living Insects

Planthoppers, leafhoppers and free-living parasitoids were collected from the rice plots using sweep-nets at 30 DAT. During sampling, ten sweeps of an entomological sweep net (rim diameter = 45 cm) were taken at each subplot (2 varieties × 2 nitrogen levels × 6 replicates × 2 seasons = 48 samples). The collected insects were transferred to 70% alcohol and were cleaned in the laboratory under a stereo microscope (Nikon SMZ-2B, Tokyo, Japan). Planthopper, leafhopper and egg-parasitoid species were identified to species or genus (parasitoids) and counted.

### 2.5. Parasitism of Planthopper and Leafhopper Eggs

In order to quantify parasitism rates under different nitrogen levels, IR62 and IR64 plants infested with planthopper or leafhopper eggs were exposed in the experimental plots. The IR64 and IR62 plants were initially sown to seed beds in a screenhouse and, at 28 DAS, were individually transplanted into pots of 5 × 5 cm (H × diam) with zero-added nitrogen or with nitrogen equivalent to 150 kg ha^−1^. The pots were individually covered within mylar cages of 45 × 10 cm (H × diam), each with a mesh top. At 50 DAS, five female and five male BPH, WBPH or GLH from the colonies were placed into the cages (each plant with a single herbivore species). The females were allowed to lay eggs in the plants for 24 h after which the adults were removed. Three plants of each variety, each plant with eggs of one of the herbivore species, were exposed in the field subplots each season (i.e., 3 species × 2 varieties × 2 nitrogen levels × 6 replicates = 72 plants per season). The potted plants were placed in subplots with the corresponding variety and nitrogen level. A set of not field-exposed, control plants (i.e., 3 insect species × 2 varieties × 2 nitrogen levels × 5 replicates = 60 pots) were maintained in a screenhouse. The control plants were used to verify that eggs were not parasitized before transfer to the field plots. We also used the controls to assess egg laying by each herbivore species as a response to variety and nitrogen level.

After 4 days, the field-exposed plants, and control plants that were maintained in the screenhouse were collected. The plants were removed from their pots and the roots rinsed under tap water. The roots were then wrapped in moistened paper towels. Each plant was stored in a test tube of 200 × 25 mm (H × diam). Tubes were wrapped with carbon paper and a translucent vial was placed at the top of each tube to attract the hatched insects to the light (i.e., to the vial). Fourteen days after collection, the tubes with the plants were placed in a freezer at −20 °C. The plant samples were dissected under a stereo microscope (Nikon SMZ-2B, Tokyo, Japan) and the numbers of eggs, hatched herbivores and parasitoids (free-living adults and parasitized herbivore eggs) were recorded. Parasitoid species were differentiated by the color of the eggs: orange eggs = *Gonatocerus* spp., reddish-brown eggs = *Anagrus* spp., yellow eggs = *Oligosita* spp.; white colored eggs were regarded as unparasitized (leafhopper and planthopper eggs). This color-based identification method is regarded as effective for assigning a genus to the parasitoids, but not for determining species [55]. However, emerged adult parasitoids were identified to the species level.

### 2.6. Honeydew and Its Effects on Egg Parasitism

To compare honeydew production by BPH on IR62 and IR64, we first conducted a greenhouse experiment. Plants (IR62 or IR64) were sown to pots of 5 × 5 cm (H × diam) with zero-added nitrogen or with nitrogen equivalent to 150 kg ha^−1^. At 14 DAS, plastic chambers (5 × 10 cm: H × diam) were placed around the base of each plant. Each chamber had a hole at the base and at the top, through which the plant passed, with a cotton plug used to seal the top hole around the plant [28]. Filter paper (Whatman No. 1) treated with bromocresol green, was placed at the base of each chamber. Bromocresol green indicates honeydew as blue-rimmed, alkaline spots and white-rimmed, acidic spots. The blue spots represent phloem-derived honeydew and the white spots, xylem-derived honeydew [30]. At 15 DAS, a single, pre-starved (2 h) gravid brachypterous female was confined to each chamber. The planthoppers were allowed to feed for 24 h after which the filter papers were collected and photographed with a Nikon D90 (12.3 megapixel) digital camera (Nikon Corp., Tokyo, Japan) on an illuminated bench. The areas of all spots were measured from the images using Image-J version 1.48 (National Institute of Health, Bethesda, MD, USA). The bioassay was replicated five times (i.e., 2 varieties × 2 nitrogen levels × 5 replicates = 20 pots).

We assessed the influence of honeydew from the different varieties on parasitism in the field plots. Sentinel plants infested with BPH eggs were prepared as described above. After the adult BPH had been removed, honeydew was removed from the plant surfaces by rinsing and wiping the plants with a wet paper towel. Plants were divided into three groups. The first group had honeydew collected from BPH feeding on the same variety applied to the stem surface of each plant (e.g., host plant = IR62, honeydew = IR62). A second group of plants had honeydew collected from BPH feeding on a different variety applied to the stem surface (e.g., host plant = IR62, honeydew = IR64). The honeydew that was applied to the plants was collected by attaching parafilm sachets [29] of 5 × 5 cm (H × W) with five gravid females inside, to plants of either IR62 or IR64 under each nitrogen regime. After 36 h, the honeydew was removed from the parafilm using micropipettes and was applied to the stem surface of the sentinel plants. During the dry season, 12 µL of honeydew was applied to each plant; during the wet season, 10 µL of honeydew was applied. The honeydew was dripped over the base of the plants and allowed to air dry. A third group of plants had no honeydew applied to the surfaces (i.e., honeydew was wiped off). Plants (with and without honeydew) were then exposed in the corresponding subplots (i.e., matching variety and nitrogen level). After 4 days, the plants were collected and processed as described above. The experiment was replicated 6 times each season (i.e., 3 treatments [with honeydew from planthoppers on the same variety, with honeydew from planthoppers on a different variety, and without honeydew] × 2 varieties × 2 nitrogen levels × 6 replicates = 72 exposed plants per season).

### 2.7. Statistical Analyses

The relative abundance of BPH, WBPH, or GLH and of the free-living stages of egg parasitoids, as well as parasitism rates estimated using sentinel plants with different herbivore eggs or under different honeydew treatments were analyzed using Generalized Linear Mixed Models (GLMM) with Template Model Builder (TMB) or Generalized Linear Mixed-Effects Models (GLMER) adequate for the split-plot field design. In cases of overdispersion, the beta-binomial distribution was fitted to the GLMMTMB models. After checking for overdispersion and randomness of the residuals, the most suitable models were selected according to the value of the Akaike Information Criterion (AIC) for each model. Parasitism rates were estimated as the number of parasitized eggs divided by the total numbers of emerged nymphs and the total number of parasitized eggs (including all parasitoids). This eliminated from the calculations possible non-viable eggs, or eggs consumed by predators. Because parasitism by different species is contemporaneous, we assessed the effects of calculating parasitism using only available eggs for corresponding species (e.g., emerged planthoppers and eggs parasitized by *Anagrus* spp. as the denominator for the calculation of *Anagrus* spp. parasitism rates). Calculations based on available eggs increased calculated parasitism rates, but did not affect patterns across varieties or nitrogen levels; therefore, we used viable eggs as the denominator in calculations. This assumes that parasitism by conspecific or congeneric parasitoids did not limit parasitism by any species. This also allowed us to sum parasitism rates from different parasitoid species to estimate total mortality of eggs from all parasitoids combined. We also checked for multicollinearity of parasitism rates by *Oligosita* spp. and *Anagrus* spp., this examination of the data also found no correlations; therefore, egg parasitism by these two species was analyzed separately. Total honeydew production and the proportions of xylem-derived honeydew were examined using Generalized Linear Models (GLM) with a Gaussian and Binomial distribution, respectively. All data were analyzed using R v4.0.2 [56].

## 3. Results

### 3.1. Arthropod Community

**Figure 1:**
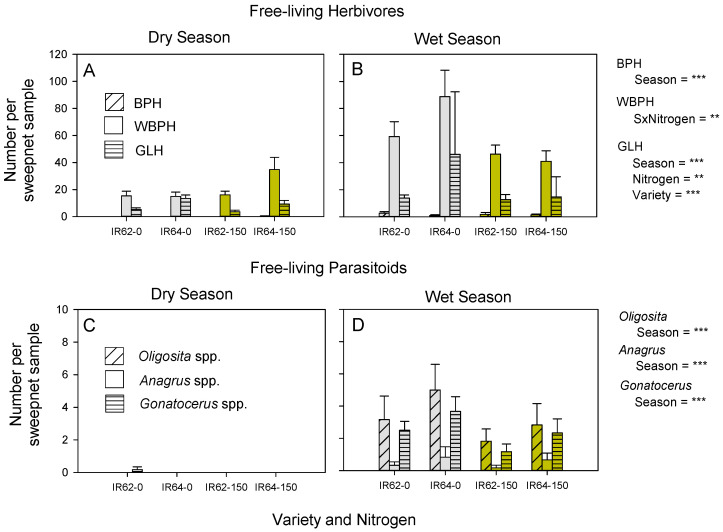
Numbers of planthoppers (**A**,**B**), leafhoppers (**A**,**B**) and free-living egg parasitoids (**C**,**D**) collected at plots of resistant (IR62) and susceptible (IR64) rice under low (gray bars: 0 added) and high (yellow bars: 150 kg N ha^−1^) levels of nitrogenous fertilizer. Sampling was conducted using sweep nets during the 2013 dry (**A**,**C**) and wet (**B**,**D**) seasons. Standard errors are indicated (N = 6); note differences in scales; results of GLM are indicated as ** = *p* ≤ 0.05 and *** = *p* ≤ 0.001; BPH = brown planthopper, WBPH = whitebacked planthopper, and GLH = green leafhopper; S = season.

Planthoppers and leafhoppers were more abundant during wet season sampling compared to the dry season (BPH: *χ^2^* = 21.656, *p* < 0.001; WBPH: *χ^2^* = 46.985, *p* < 0.001; GLH: *χ^2^* = 29.568, *p* < 0.001: Figure 1A,B). WBPH was the most abundant herbivore. There was no apparent variety effect on BPH (*χ^2^* = 0.364, *p* > 0.05) or WBPH (*χ^2^* = 1.392, *p* > 0.05) abundance in the field plots; however, more GLH (*χ^2^* = 20.838, *p* < 0.001) were captured in sweep nets at the IR64 plots. More GLH (*χ^2^* = 7.102, *p* < 0.01) were captured in low- than in high-nitrogen plots (Figure 1A,B); but captures of BPH were not affected by nitrogen level (BPH: *χ^2^* = 0.058, *p* > 0.05) (Figure 1A,B). There was a significant season–nitrogen interaction for WBPH (*χ^2^* = 8.196, *p* < 0.01) because of higher captures in low-nitrogen plots than in high-nitrogen plots during the wet season, but not the dry season (Figure 1A,B). 

Egg parasitoids were also more abundant during wet season sampling (*Oligosita* spp.: *χ*^2^ = 72.667, *p* < 0.001; *Anagrus* spp.: *χ*^2^ = 9.075, *p* < 0.01; *Gonatocerus* spp.: *χ*^2^ = 84.525, *p* < 0.001: Figure 1C,D). *Oligosita* spp. (mainly *O.* sp. nr *aesopi*) and *Gonatocerus* spp. (mainly *G.* sp. nr. *orientalis*) were the most abundant egg parasitoids. At least two *Anagrus* spp. (including *A. flaveolus* and *A. optabilis*) occurred at relatively low numbers in the plots (Figure 1C,D). More parasitoids were captured in low-nitrogen plots; however, there was no statistically significant effect of nitrogen (3.088 ≥ *χ*^2^ ≥ 0.142, *p* ≥ 0.05) or variety (0.740 ≥ *χ*^2^ ≥ 0.344, *p* ≥ 0.05) on captures of any of the three parasitoid groups (Figure 1C,D). 

### 3.2. Parasitism of Planthopper and Leafhopper Eggs

Egg laying by planthoppers was not affected by variety and was consistent between the two seasons that we used sentinel plants (Table S1). BPH laid 100.15 ± 4.03 eggs per plant; WBPH laid 65.92 ± 5.25 eggs per plant. All three hoppers tended to lay more eggs in nitrogen-fertilized plants, but the effects were only significant for GLH (*χ*^2^ = 8.372, *p* < 0.01). During our experiments, GLH laid significantly fewer eggs during the wet season experiments (DS = 101.53 ± 15.00; WS = 19.93 ± 3.75: *χ*^2^ = 62.987, *p* < 0.001) (Table S1). Eggs maintained in the screenhouse were not parasitized, indicating that all reported parasitism occurred during field exposures.

**Figure 2:**
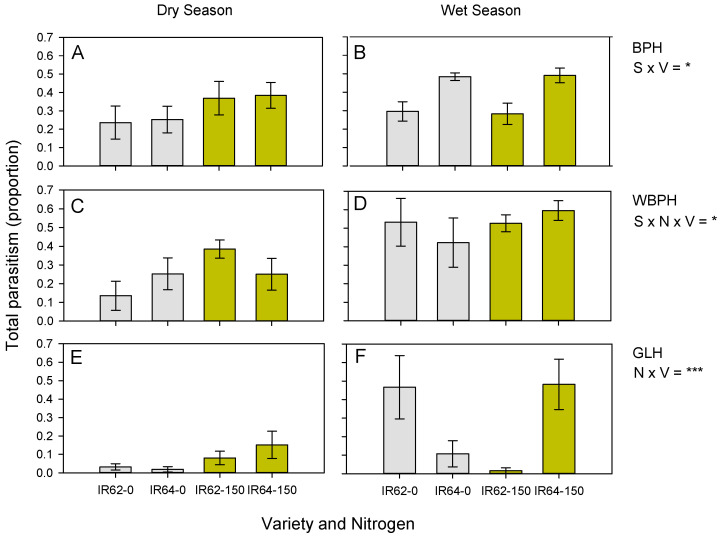
Effects of nitrogen (0 or 150 kg ha^−1^ of nitrogen) and variety (IR62: resistant and IR64: susceptible) on percentage parasitism of BPH (**A**,**B**), WBPH (**C**,**D**) and GLH (**E**,**F**) eggs in rice field plots. Experiments were conducted during the dry (**A**,**C**,**E**) and wet (**B**,**D**,**F**) seasons of 2013. Standard errors are indicated (N = 6); results of GLM are indicated as ns = *p* > 0.05, * = *p* ≤ 0.05 and *** = *p* ≤ 0.001. BPH = brown planthopper, WBPH = whitebacked planthopper, and GLH = green leafhopper; S = season, N = nitrogen and V = variety.

Egg parasitoids caused 31.04 ± 4.01% mortality of BPH eggs during the dry season and 38.88 ± 2.96% during the wet season exposures. There was no effect of nitrogen (*χ*^2^ = 3.110, *p* > 0.05) on parasitism levels. There was a significant season–variety interaction (4.293, *p* = 0.038) because of higher parasitism of eggs in IR64 than IR62 during the wet season exposure, but not during the dry season (Figure 2A,B).

Parasitism of WBPH eggs was lower in the dry season (23.63 ± 3.98%) than the wet season (51.96 ± 4.81%) (*χ*^2^ = 12.666, *p* < 0.001: Figure 2B,C). There was a tendency toward higher parasitism of WBPH eggs in high-nitrogen plots. However, the three-way [season–variety–nitrogen] interaction was significant (*χ*^2^ = 4.871, *p* = 0.027) (Figure 2C,D). 

Parasitism of GLH eggs was low during the dry season exposure (7.08 ± 2.23%), with a non-significant increase during the wet season exposure (26.83 ± 6.71%: *χ*^2^ = 1.607, *p* = 0.209) (Figure 2E,F). *Oligosita* spp. (mainly *O.* sp. nr *aesopi*) were the main parasitoids. In both seasons, egg parasitism was higher on IR62 plants under low nitrogen, but higher on IR64 plants under high nitrogen (*χ*^2^ = 15.852, *p* < 0.001: Figure 2C,D). We will not consider parasitism of GLH eggs further in this study because of low parasitism rates during the dry season exposure and low numbers of eggs laid in sentinel plants in the wet season (Table S1). 

**Figure 3:**
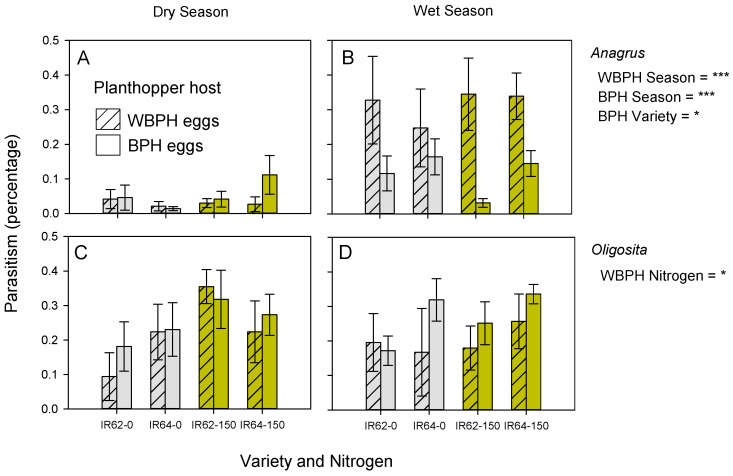
Effects of nitrogen (0 or 150 kg ha^−1^ of nitrogen) and variety (IR62: resistant and IR64: susceptible) on parasitism of WBPH and BPH eggs by *Anagrus* spp. and *Oligosita* spp. in field plots. Experiments were conducted during the dry (**A**,**C**) and wet (**B**,**D**) seasons of 2013. Standard errors are indicated (N = 6); results of GLMs are indicated as * = *p* ≤ 0.05 and *** = *p* ≤ 0.001. BPH = brown planthopper and WBPH = whitebacked planthopper.

Parasitism rates of WBPH (*χ*^2^ = 32.814, *p* < 0.001) and BPH (*χ*^2^ = 13.168, *p* < 0.001) eggs by *Anagrus* spp. were significantly higher during the wet season compared to the dry season exposure (*χ*^2^ = 13.168, *p* < 0.001: Figure 3A,B). A higher proportion of BPH eggs were parasitized on IR64 compared to IR62 (*χ*^2^ = 6.527, *p* < 0.01), but the same variety effect did not occur for WBPH eggs (*χ*^2^ = 0.570, *p* > 0.5: Figure 3A,B). 

Parasitism of WBPH eggs by *Oligosita* spp. was marginally higher in the high-nitrogen plots (*χ*^2^ = 4.454, *p* < 0.05), but the same effect did not occur for BPH eggs (*χ*^2^ = 0.389, *p* > 0.05: Figure 3C,D). Egg parasitism by *Oligosita* spp. was not affected by season, variety or interactions during sampling (Figure 3C,D).

### 3.3. Honeydew and Its Effects on Parasitism

BPH produced less honeydew when feeding on IR62 compared to IR64 and honeydew produced on IR62 was mainly derived from xylem feeding (Table 1).

**Figure 4:**
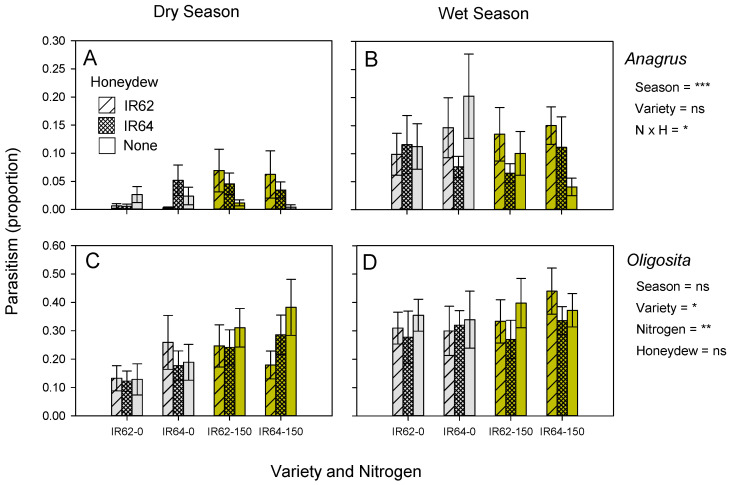
Parasitism of BPH eggs by *Anagrus* spp. (**A**,**B**) and *Oligosita* spp. (**C**,**D**) on plants with one of three honeydew treatments (IR62-derived honeydew added = hatched bars; IR64-derived honeydew added = cross-hatched bars; honeydew removed = open bars). Sentinel IR62 (resistance) and IR64 (susceptible) plants with BPH eggs were exposed in field plots with low (0 added) and high (150 kg ha^−1^) nitrogenous fertilizer during the dry (**A**,**C**) and wet (**B**,**D**) seasons of 2013. Standard errors are indicated (N = 6); results of GLMs are indicated as ns = *p* > 0.05, * = *p* ≤ 0.05, ** = *p* ≤ 0.01 and *** = *p* ≤ 0.001.

Parasitism of BPH eggs by *Anagrus* spp. was relatively low during the dry season exposure but higher during the wet season exposure (*χ*^2^ = 24.833, *p* < 0.001). There was a significant nitrogen–honeydew interaction (*χ*^2^ = 8.558, *p* = 0.014) because of higher parasitism by *Anagrus* spp. of BPH eggs on plants with IR62-derived honeydew than on plants without honeydew in high-nitrogen plots (irrespective of variety), but similar parasitism levels on plants with different honeydew treatments in low-nitrogen plots (Figure 4A,B).

Parasitism of BPH eggs by *Oligosita* spp. was similar during the dry and wet season exposures (*χ*^2^ = 2.085, *p* = 0.149). A higher proportion of eggs were parasitized in IR64 plants (*χ*^2^ = 5.986, *p* = 0.014) and under high nitrogen (*χ*^2^ = 8.522, *p* = 0.004). Honeydew treatments had no significant effect on parasitism by *Oligosita* spp. (*χ*^2^ = 5.010, *p* = 0.082) (Figure 4C,D). The covariate ‘egg density’ significantly, positively influenced parasitism rates (*χ*^2^ = 77.133, *p* < 0.001).

## 4. Discussion

During our experiments, parasitism by *Oligosita* spp. was often higher in field plots under high nitrogen (Table 2). Rice plant growth rates increase under high-nitrogen conditions and plants under high nitrogen are more susceptible to a range of pests, particularly sap-sucking pests [4,6,31,36]. Although we did not quantify or qualify volatiles emitted from rice under low or high nitrogen, the higher parasitism by *Oligosita* spp., despite relatively constant egg numbers in exposed trap plants (Table S1), suggests that eggs in high-nitrogen plants were more attractive to the parasitoids. This supports the Optimal Defense Hypothesis, and indicates a possible positive feedback mechanism contributing to the regulation of planthopper populations in the rice crop. Parasitism of eggs in IR64 (susceptible) by *Oligosita* spp. and *Anagrus* spp. was also sometimes higher than eggs in IR62 (resistant) (Table 2). This further suggests that parasitism was directed to the more vulnerable plants, and, because of relatively constant densities of eggs in those plants during our experiments (Table S1), that the effect was probably mediated by semiochemicals. For *Oligosita* spp., the effect was independent of the presence or composition of honeydew on the plants (Table 2); however, for *Anagrus* spp., there was evidence that honeydew is associated with higher parasitism rates, and the effect was most apparent for IR62-derived honeydew under high-nitrogen conditions (Table 2). This represents a further feedback mechanism that directs parasitoids to resistant rice plants when these are most vulnerable to planthopper attack (Table 2). We discuss these results in the context of the multiple factors influencing parasitism rates in rice field plots.

### 4.1. Variety and Nitrogen Effects on Planthopper and Leafhopper Abundance

Several previous studies of IR62 have indicated that the variety has strong antixenosis and antibiosis defenses [49,54,57]. In greenhouse and field cages, the build-up of BPH populations on IR62 is curtailed over successive generations and populations may eventually collapse, even in the absence of natural enemies [58]. Our experiments were conducted on relatively young plants because maximum egg laying by BPH and WBPH occurs in young plants [59] and egg parasitoids are most abundant at early crop stages [38,60]. We found no significant effect of variety on egg laying by either planthopper species, or by GLH (Table S1); however, recent studies have shown that egg laying by BPH under no-choice conditions is heavily affected by the host of the female parent (i.e., TN1 in this study), with reductions in egg laying apparent as a lack of successive oviposition cycles [61]. The success of IR62 therefore relies on effective suppression, or losses in the reproductive potential of early generations of nymphs produced by immigrating females, which occurs during early crop stages. Variety effects may be more apparent at later crop stages, but these effects are initiated in the early crop. Therefore, a lack of apparent variety effects on planthoppers in our field plots (Figure 1A,B) may be due to the early crop stage at which sampling was conducted, but could also be due the low captures (BPH), and only late-stage resistance in IR62 to WBPH [31]. In contrast to planthoppers, GLH was, as expected, less abundant in plots of IR62 (Figure 1A,B).

A number of studies have shown that the fitness (survival × reproduction) of BPH, WBPH and GLH increases under high nitrogen [54,59,62,63]. Based on these reports, we expected that planthopper and leafhopper densities would be higher in high-nitrogen plots; however, sweep-net sampling indicated that GLH were consistently more abundant in the low-nitrogen plots (Figure 1A,B); WBPH were also more abundant in low-nitrogen plots during the wet season (Figure 1B). These results agree with results from previous sampling at the same plots in different years as reported by Horgan et al. (2019) [22]. The lower abundance of WBPH and GLH in low-nitrogen plots, despite clear benefits of high nitrogen for these species, suggests that mortality is higher in high-nitrogen plots. Outbreaks of BPH have been associated with excessive fertilizer use [4,64]; however, nitrogenous fertilizer had no apparent effect on BPH numbers in our study (Figure 1A,B). A lack of apparent effects in our study may be because the species occurred at low densities (generally below economic threshold densities), and, as with variety effects (explained above), the effects of nitrogen may be more apparent during later crop stages when second- or third-generation planthoppers occur [4,5]. Nevertheless, our study did find significant variety and nitrogen effects on egg parasitism.

### 4.2. Nitrogen Effects on Egg Parasitoids

Hymenopteran parasitoids cause significant mortality to planthopper eggs [65,66,67]. Using field cages in Indonesia that excluded different components of arthropod natural enemy communities, Claridge et al. (2002) [65] estimated that egg parasitoids reduced BPH populations by approximately 50%, with predatory bugs and spiders further reducing populations to below 1% of their potential. Furthermore, a number of studies have indicated that egg parasitoids respond to egg densities at plant and batch scales [66,68,69]. In the present study, egg parasitism by *Oligosita* spp. was also density dependent at the plant scale (Table 2). Adult *Oligosita* spp. and *Gonatocerus* spp. occurred in higher numbers at low-nitrogen plots, where phloem-feeding herbivores were also more abundant (Figure 1D). However, the parasitism of eggs in sentinel plants was generally higher in high-nitrogen plots (i.e., *Oligosita* spp. with WBPH—Figure 3; *Oligosita* spp. with BPH—Figure 4; *Anagrus* spp. with BPH—Figure 4). Discrepancies between the results of sweep-net sampling and sentinel plants are probably due to the diversity of *Oligosita*, *Anagrus* and *Gonatocerus* species in the rice ecosystem. For example, *Gonatocerus* spp. were relatively common in sweep nets in the wet season, but egg parasitism by *Gonatocerus* was low (i.e., <1%) each season, suggesting that most of the *Gonatocerus* spp. captured in sweep nets were parasitoids of arthropod eggs other than BPH, WBPH or GLH.

Barrion et al. (1981) [70] identified several egg parasitoids of planthopper and leafhopper eggs in Luzon that included *Mymar tapobanicum* (Ward) and *Polynema* sp. (Mymaridae), and *Stephanodes* sp. (Trichogrammatidae) that we did not detect among parasitized eggs. Furthermore, Sann et al. (2018) [71] have shown that the diversity of *Anagrus* and *Gonatocerus* species that parasitize BPH and GLH (and presumably WBPH) in the Philippines is underestimated because of the occurrence of several cryptic species. Because the results from sentinel plants are directly related to parasitoid function, our results indicate that egg parasitism by *Oligosita* spp. in particular is higher under high-nitrogen conditions (Figure 2, Figure 3 and Figure 4) and therefore likely stabilizes nitrogen-induced increases in planthopper populations. Although the number of WBPH eggs in sentinel plants was not statistically significantly affected by nitrogen content (Table S1), slightly higher numbers in the high-nitrogen plants, together with apparent density dependent parasitism [66,68,69], may have contributed to the higher rates of parasitism estimated for high-nitrogen plots; however, the association was not apparent in all experiments (Table 2), thereby suggesting that semiochemical lures also contributed to the effect in all cases. Because *Oligosita* spp. were the most abundant parasitoids and are responsible for higher parasitism rates compared to other parasitoids (i.e., *Anagrus* spp.) in Philippine rice fields, and based on evidence for the involvement of semiochemical lures, then our results support the Optimal Defense Hypothesis.

### 4.3. Variety Effects on Egg Parasitoids

Unlike the parasitoids of free-living herbivore stages, egg parasitoids must encounter immobile life-stages that do not, by themselves, damage the host plant [33]. In the case of BPH, WBPH and GLH, the eggs are further concealed beneath the host parenchyma [55]. Egg parasitoids therefore rely on a range of chemical cues to locate their hosts. These are mainly induced volatiles emitted from herbivore damaged plants [37,72,73,74]. For egg parasitoids, volatile-inducing damage is predominantly related to feeding activities of the adult planthoppers or leafhoppers, although parasitoids will also respond to cues emitted after attack by planthopper nymphs [74]. Much recent research has focused on identifying the volatiles emitted from BPH damaged rice plants [37,75,76]. However, there is still little knowledge of the chemical ecology of egg parasitism. Lou et al. (2005) [74] have shown that the egg parasitoid *Anagrus nilaparvatae* Pang et Wang is attracted to rice plants on which BPH are feeding; several plant volatiles appear to play a role in this attraction. Furthermore, rice plants treated with jasmonic acid (JA) have been shown to emit a range of volatiles associated with the attraction of *A. nilaparvatae*, and BPH eggs in rice plants that are surrounded by JA-treated plants are more vulnerable to the egg parasitoid [73]. However, Lou et al. (2006) [37] have also shown that the range and number of volatiles emitted from rice are strongly influenced by variety. In a comparative study, *A. nilaparvatae* parasitized fewer eggs on IR26 and IR64, both varieties with the *Bph1* gene for resistance, compared to eggs in susceptible varieties without BPH resistance genes [37]. Parasitism was also generally higher in JA-treated susceptible plants compared to JA-treated resistant plants [37]. Volatile blends therefore strongly influence parasitoid attraction to infested plants.

We predicted that parasitism of eggs in IR64 would be higher than in IR62. We based our predictions on previous research by Lou and colleagues [37] that suggested resistant plants may be less attractive to egg parasitoids, and on the likely lower densities of eggs on resistant rice. However, we also expected that IR62, which has high antibiosis defenses, would either have a lower requirement for semiochemical lures, compared with susceptible varieties, or would release fewer volatiles during attack because of resistance-induced changes in planthopper feeding behaviors. In support of our predictions, we found that total parasitism of BPH eggs was higher on IR64 than IR62 during the wet season exposures (Figure 2, Table 2). Furthermore, parasitism of BPH eggs by *Anagrus* spp. was higher on IR64 than on IR62 (Figure 3A,B, Table 2), with a tendency toward higher *Oligosita* spp. parasitism on IR64 in the same experiment (Figure 3D), and significantly higher *Oligosita* spp. parasitism on eggs in IR64 in the honeydew experiment (Figure 4, Table 2). Parasitism of WBPH eggs by *Oligosita* spp. also tended to be higher on IR64 (Figure 3). As discussed previously, higher parasitism of eggs in IR64 cannot be attributed to density effects at the plant level (Table S1). It is more probable that the differences are associated with the dissimilar behaviors of planthoppers on the varieties. As indicated in a previous study, whereas BPH oviposit similarly on IR62 and susceptible varieties during initial oviposition cycles, the feeding behavior of adult females on resistant and susceptible varieties differs considerably [61]. Planthoppers on resistant rice will probe more than on susceptible rice, they also have shorter feeding bouts and produce less honeydew [77]. Each of these factors could influence the nature of volatiles emitted from infested rice plants and ultimately affect egg parasitism. We examined the potential for one of these possible factors, honeydew composition, to influence parasitism rates.

### 4.4. The Role of Honeydew in Egg Parasitism

We found that the BPH on IR62 produced less honeydew and the honeydew had higher proportions of acidic xylem-derived materials than honeydew produced on IR64. Honeydew is a sweet and sticky substance that contains sugars [78]. However, the honeydew of planthoppers also contains a range of other chemicals including proteins of insect, plant and bacterial origin [79], plant hormones, including feeding inhibitors [80], and plant defense elicitors of insect or microbial origin [76,79]. BPH honeydew has recently been shown to also contain microbial symbionts that enhance rice defenses against insects and diseases, which may ultimately induce rice volatiles that attract natural enemies [76]. Honeydew is a rich source of nutrients for the insects that consume it [34,45,81]. Indeed, recent evidence suggests that honeydew is often the main food source for aphid parasitoids even in the presence of diverse nectar sources [35]. Furthermore, honeydew may include host-location kairomones or function as a stimulator of oviposition for parasitoids [32,45,82].

We found no evidence that honeydew plays a role in the attraction of *Oligosita* spp to infested plants (Figure 4C,D, Table 2). However, honeydew did play a role in parasitism by *Anagrus* spp. (Table 2). We found that egg parasitism by *Anagrus* spp. was higher on plants with honeydew than on plants without honeydew; however, the effect was only apparent for plants in high nitrogen pots/plots. Because *Anagrus* spp. parasitized more eggs on IR64 (Figure 3A,B), we expected that honeydew from IR64 would also be most attractive to the species. However, the parasitism of eggs in high-nitrogen plants (either IR62 or IR64) with IR62 honeydew was consistently higher than in high-nitrogen plants with IR64 honeydew (Figure 4A,B). However, in our experiments, we used equal amounts of honeydew on susceptible and resistant plants; under natural conditions, the quantities of honeydew on IR62 plants would be considerably lower than on IR64 plants (Table 1)–which may explain why *Anagrus* spp. parasitized more eggs in the IR64 plots. *Anagrus* spp. may also prefer plants based on the availability of nutrients in the associated honeydew, as suggested by the relatively strong nitrogen effect in the experiment (Figure 4A,B, Table 2).

The mechanisms leading to higher parasitism on plants treated with IR62-derived honeydew compared to equal amounts of IR64-derived honeydew are difficult to suggest. Parasitoid behaviors are complex and it may be that IR62-derived honeydew provides nutrients to parasitoids in a more diluted form (because xylem-derived honeydew contains more water) possibly avoiding the need to clean mouth and body parts [78] and reducing searching and handling times. IR62 honeydew may also have higher levels of JA, specific volatiles, or sucking inhibitors that directly or indirectly attract parasitoids. As indicated by Luo et al. (2006) [37], the nature of volatiles emitted from rice plants is difficult to predict and the volatile blends emitted are not grouped according to resistance levels. Future studies that compare several resistant and susceptible varieties for their honeydew-related effects on parasitism are required to better understand these complex interactions and to test general hypotheses concerning egg parasitism in crops of resistant rice. However, our results do indicate that *Anagrus* spp. respond to honeydew in high-nitrogen plots, and that honeydew from resistant rice is not necessarily unattractive or otherwise less beneficial to egg parasitoids. Furthermore, higher egg parasitism associated with IR62-derived honeydew (Figure 4A,B), when these plants become more vulnerable under high soil nitrogen regimes, may compensate for the normally low attraction of parasitoids to resistant IR62 plants (Figure 2).

### 4.5. Incorporating Resistance into Agroecological Designs for Rice Ecosystems

To our knowledge, only one study has examined the combined effects of host resistance, nitrogen inputs, and conservation biological control on rice planthoppers and leafhoppers [22]. In that study, using the same platform as in the present study, researchers found that planting bunds with mung bean (*Vigna radiata* (L.) R. Wilczek) was associated with higher numbers of *Oligosita* spp. in sweep-net samples from adjacent rice; the effects were stronger when planted bunds were adjacent to high-nitrogen plots [22]. Meanwhile, bunds planted with mung bean had no significant effect on the abundance of *Anagrus* spp. parasitoids, but parasitism of eggs in high-nitrogen plots was higher where the plots were adjacent to bunds planted with sesame (*Sesamum indicum* L.) or where bund weeds had not been removed [22]. In greenhouse studies, Zhu et al. [26] indicated that sesame in particular is associated with improved parasitism rates of *A. nilaparvatae*. In a further field study, Vu et al. (2018) [24] found that parasitism of BPH eggs in sentinel rice plants declined at increasing distances from patches of lady finger (*Abelmoschus esculentus* (L) Moench) and into rice fields. The effect was largely due to *Oligosita* spp. egg parasitism. The role of non-rice habitat for egg parasitoids is now well established, particularly for *Anagrus* spp. (mainly *A. nilaparvatae*) in China, and *Oligosita* spp. in the Philippines [20,22,26,83,84]. In our system, *Oligosita* spp. were generally abundant and are probably more mobile in rice production landscapes, whereas *Anagrus* spp. appear to be rice field specialists. Our results suggest that the main *Anagrus* spp. parasitoids in our study system are also affected by the availability and quality of honeydew coating infested rice plants. This may explain why bund vegetation seems to affect *Anagrus* spp. less than *Oligosita* spp. [22] and why *Anagrus* spp. are more severely affected by insecticides compared to *Oligosita* spp. [69]. Recent studies have shown that honeydew can be contaminated by pesticides [85], which may increase the vulnerability of species such as *Anagrus* spp. to insecticide treatments (but see Heinrichs et al. 1982 [67]).

## 5. Conclusions

Egg parasitism is a significant source of mortality that contributes to the regulation of BPH, WBPH and possibly GLH populations in rice. Egg parasitism is generally greater in high-nitrogen field plots, representing a potential constraint on the nitrogen-induced enhancement of planthopper and leafhopper fitness and supporting the Optimal Defense Hypothesis. Two abundant parasitoid groups, *Oligosita* spp. and *Anagrus* spp., are active in Philippine rice fields. The abundance of *Oligosita* spp. in rice fields and consequent egg parasitism have been shown to increase close to vegetation patches [22]. Meanwhile, *Anagrus* spp. respond to honeydew produced by planthoppers on high-nitrogen rice plants through increased egg parasitism rates. Parasitism of eggs was frequently higher on IR64 (susceptible) than IR62 (resistant) rice plants; but a preference for honeydew from IR62 suggests that *Anagrus* spp. work in synergy with the resistant variety to dampen planthopper population responses to high nutrient availability. This effect could prolong the durability of resistant rice against emerging virulent planthopper and leafhopper populations.

## Figures and Tables

**Table 1:** Honeydew production by BPH on IR64 (susceptible) and IR62 (resistant) rice plants. Numbers are means ± SEM (N = 5).

Honeydew Production	Variety and Nitrogen				*χ*^2^-Values ^1^	
	IR62		IR64		Variety	Nitrogen
	0 Added N	150 kg N ha^−1^	0 Added N	150 kg N ha^−1^		
Total (mm^2^)	0.31 ± 0.08	0.44 ± 0.09	1.20 ± 0.07	0.99 ± 0.15	51.173 ***	0.186 ns
Xylem Derived (proportion)	0.85 ± 0.10	0.65 ± 0.10	0.10 ± 0.03	0.20 ± 0.08	38.908 ***	0.284 ns

^1^: ns = *p* > 0.05; *** = *p* ≤ 0.001; variety–nitrogen interactions were non-significant.

**Table 2:** Summary of results from field experiments on the effects of variety (IR62= resistant or IR64 = susceptible), nitrogenous fertilizer level (low = 0 kg N ha^−1^ or high = 150 kg N ha^−1^), honeydew (IR62-derived, IR64-derived, or no honeydew) and their interactions on parasitism of planthopper and leafhopper eggs.

Herbivores ^1^	Parasitoids	Variety Effect ^2^	Nitrogen Effect ^2^	Season Effect ^1,2^	Interaction Effects ^2^	Egg Density (Covariate) ^2^
Abundance in sweep-nets						
BPH		ns	ns	WS ***	ns	
WBPH		ns	-	-	Low nitrogen × WS **	
GLH		IR64 ***	Low **	WS ***	ns	
	*Oligosita* spp.	ns	ns	WS ***	ns	
	*Anagrus* spp.	ns	ns	WS **	ns	
	*Gonatocerus* spp.	ns	ns	WS ***	ns	
Parasitism of eggs in sentinel plants						
BPH	All combined	-	ns	-	IR64 × WS *	ns
WBPH	All combined	-	-	-	Variety × nitrogen × season *^,3^	ns
GLH	All combined	-	-	ns	IR62 × low nitrogen; IR64 × high nitrogen ***	ns
BPH	*Oligosita* spp.	ns	ns	ns	ns	ns
WBPH	*Oligosita* spp.	ns	High *	ns	ns	ns
BPH	*Anagrus* spp.	IR64 **	ns	WS ***	ns	ns
WBPH	*Anagrus* spp.	ns	ns	WS ***	ns	ns
Parasitism of eggs in sentinel plants (honeydew)						
BPH	*Oligosita* spp.	IR64 **	High ***	ns	ns	***
BPH	*Anagrus* spp.	-	ns	WS ***	IR62-derived honeydew × high nitrogen **	ns

^1^: BPH = brown planthopper, WBPH = whitebacked planthopper, GLH = green leafhopper, and WS = wet season; ^2^: ns = *p* > 0.05, * = *p* ≤ 0.05, ** = *p* ≤ 0.01, *** = *p* ≤ 0.001; ‘-’ = factor involved in significant interaction; levels associated with highest proportions of eggs parasitized are indicated for each significant factor; ^3^: levels are not highlighted for significant three-way interactions.

## Data Availability

The data presented in this study are available on reasonable request from the corresponding author.

## Supplementary Materials

The following supporting information can be downloaded at: https://www.mdpi.com/article/10.3390/insects13030251/s1. Table S1: Oviposition by planthoppers and leafhoppers on IR64 (susceptible) and IR62 (resistant) sentinel rice plants grown in pots at low (0 added N) and high (150 kg N ha^−1^) nitrogen.
