# Supplementary material for: Interactions between Rice Resistance to Planthoppers and Honeydew-Related Egg Parasitism under Varying Levels of Nitrogenous Fertilizer
Source: Insects. 2022 Mar 1;13(3):251. doi: 10.3390/insects13030251 (PMC8948641; doi:10.3390/insects13030251)
Supplement: Supplementary file 1 [file insects-13-00251-s001.zip › insects-1606398-supplementary.pdf]

Table S1. Oviposition by planthoppers and leafhoppers on IR64 (susceptible) and IR62 (resistant) sentinel rice plants grown in pots at low (0 added N) and high (150 Kg N ha-1) added nitrogen during the dry and wet seasons. Plants were maintained in a screenhouse. Numbers are means ± SEM (N = 5)

Variety and Nitrogen Level	Number of Eggs per Plant[1]		
	BPH	WBPH	GLH
**Dry season**			
IR62 - 0 added N	96.00 ± 25.66	68.00 ± 28.21	69.67 ± 6.57
IR62 - 150 Kg N ha^{-1}	122.00 ± 16.74	124.00 ± 12.06	145.33 ± 22.23
IR64 - 0 added N	64.00 ± 7.21	78.67 ± 5.04	87.67 ± 11.32
IR64 - 150 Kg N ha^{-1}	106.33 ± 18.28	142.33 ± 12.13	101.00 ± 52.51
**Wet season**			
IR62 - 0 added N	102.50 ± 24.50	51.33 ± 19.24	5.00 ± 5.00
IR62 - 150 Kg N ha^{-1}	102.33 ± 17.40	116.67 ± 58.05	10.33 ± 10.33
IR64 - 0 added N	119.67 ± 8.74	57.33 ± 44.52	12.67 ± 8.57
IR64 - 150 Kg N ha^{-1}	110.00 ± 17.62	70.33 ± 30.07	18.00 ± 7.55
Variety (V)[2]	0.366ns	0.438ns	1.471ns
Nitrogen (N)[2]	1.235ns	2.781ns	8.372**
Season (S)[2]	1.204ns	1.152ns	62.987***

[1:] BPH = brown planthopper, WBPH = whitebacked planthopper, GLH = green leafhopper; [2:] ns = $p > 0.05$; ** = $p \leq 0.01$, *** = $p \leq 0.001$; all interactions were non-significant
